# Fall Risk Reduction Program Paired with a Transportation Program in an Underserved, Urban Minority Community: A Qualitative Evaluation

**DOI:** 10.1155/2019/2719290

**Published:** 2019-07-01

**Authors:** Thelma J. Mielenz, Laura Durbin, Fern Hertzberg, Diana Noble-Hernandez, Julie A. Sorensen

**Affiliations:** ^1^Mailman School of Public Health, Columbia University, 722 West 168th Street, New York, NY 10032, USA; ^2^ARC XVI Fort Washington Inc. (ARC), 4111 Broadway, New York, NY 10033, USA; ^3^Bassett Research Institute, One Atwell Rd., Cooperstown, NY 13326, USA

## Abstract

This study sought to evaluate A Matter of Balance/Volunteer Lay Leaders (AMOB/VLL) fall prevention curriculum in combination with a “door-through-door” program: Coordinated Older-Adult Senior Transportation Services (COASTS) for older adults living in an urban, underserved community. AMOB/VLL participants were offered eight 2-hour classes as part of the training program. Focus groups were conducted with older adult participants, COASTS mobility facilitators, and AMOB/VLL master trainers. A thematic analysis was conducted, and primary themes relating to curriculum content, cultural relevancy, and outcomes were examined. Older adults and facilitators felt the course was rewarding and led to improvements in mobility and confidence. Master trainers were more critical and recommended simplifying content, with tailored guidance for specific populations. They also recommended increased emphasis on balance and physical activity. Although participants and MoFas felt combining AMOB/VLL and COASTS was rewarding and improved participant mobility, master trainers and participants suggested minor modifications to increase program benefits for urban, underserved communities.

## 1. Introduction

Every year, one out of every four adults over the age of 65 experiences a fall [1]. Falls are the leading cause of injury-related death in this age group and can result in other negative outcomes including fear of falling (often leading to decreased mobility), loss of independence, and reduced quality of life [2–4]. The associated, direct medical costs of nonfatal falls have recently been estimated to be nearly $31.3 billion annually [5]. National Center for Health Statistics data indicate roughly three out of every four adults over 70 suffer from balance impairments [6]. Elements of the environment, such as safety, attractiveness, and site design, can also considerably impact the mobility of older adults [7], particularly for urban adults living in low SES neighborhoods who may be concerned about safety or pedestrian thoroughfares that are in disrepair. Numerous evidence-based interventions have been developed in recent years to increase fall prevention behaviors although program adoption has been poor [3].

Ross et al. [8] highlighted in a meta-analysis on falls prevention that there is a need for increased translation of evidence-based programs, such as the A Matter of Balance/Volunteer Lay Leader (AMOB/VLL) program. The AMOB/VLL program [9] focuses on cognitive-behavioral techniques to reduce fear of falling and improve outcomes associated with falling, rather than targeting falls directly [8]. Most importantly, the AMOB/VLL behavioral change approach is vital for sedentary people with limited mobility, as starting exercise programs among this population too quickly could actually increase the rate of falls [10, 11]. Overall, AMOB/VLL has been found to be effective in increasing balance confidence, physical activity, and social interaction [12–15].

In order to overcome barriers to program adoption, one potentially promising option is to improve infrastructure for delivery, such as providing transportation and mobility assistance, for older adult program participants. However, to further increase adoption and improve dissemination of programs like AMOB/VLL, assessments of curriculum content, formats, and delivery are needed. Recent evaluations of these programs have focused almost exclusively on quantitative measures of participant outcomes, and so, relatively little is known regarding participant experiences with AMOB/VLL training [12–15].

The goals of our qualitative evaluation were (1) to evaluate combining a program like AMOB/VLL with an ongoing transportation program (Coordinated Older-Adult Senior Transportation Services (COASTS) with mobility facilitators (MoFas)) and (2) to determine how the program could be altered to best serve participant needs, particularly for minorities living in underserved, urban communities.

## 2. Materials and Methods

### 2.1. General AMOB/VLL and COASTS Study Design

Starting in March 2013, the AMOB/VLL program was offered to older adults residing in the Washington Heights, New York metropolitan area, who were participating in the COASTS “door-through-door” transportation service. The Action for the Retired Community XVI Fort Washington, Inc. (ARC) senior center had previously received a grant through the Department of Transportation to provide door-through-door transportation to their older adult members with mobility disability throughout Northern Manhattan above 110^th^ Street. This service includes MoFas who assist older adults with transportation to and from locations throughout the city, such as the store, the hospital, or their local senior center. A lead trainer for AMOB/VLL trained 20 master trainers in January 2013 at Columbia University's CTSA Community Engagement Resource. Five staff members from ARC were trained as master trainers, and four of these staff taught AMOB/VLL classes for this study. These master trainers were selected based on the following eligibility criteria: (1) bilingual in English and Spanish; (2) committed to completing the AMOB/VLL training program certificate; (3) committed to conducting two coaching sessions twice (each four-hour sessions); and (4) committed to teaching two AMOB/VLL classes at Columbia University's CTSA Community Engagement Resource or ARC in Spring 2013. Less than a month later, eight additional ARC staff members were trained as coaches via the train-the-trainer model. In total, there were 12 master trainers/coaches at ARC who both completed the AMOB/VLL training program certificate and taught AMOB/VLL classes.

Upon original employment in September 2011, eight MoFas completed a mandatory week-long COASTS training module that focused on coordinated transportation organizational policies; emergency protocols and escalation; pretrip inspection; defensive driving; driver safety; passenger service and safety; older adult, low-income, and disabled sensitivity training; customer service policies and protocols; crises management containment; CPR and basic first aid; drug and alcohol policies; and Americans with Disabilities Act transportation guidelines. This training is important as the “door-*through*-door” program differs from the more traditional “door-*to*-door” programs, by providing more comprehensive assistance to seniors. For example, if needed, the COASTS MoFas can provide assistance out of the older adult's home, into and out of the vehicle, through the door of the selected destination, and then back again to the older adult's home [16]. This first training was supplemented for the purposes of our study with two additional four-hour, in-service trainings (provided by Diana Noble-Hernandez) based on the *Fall Prevention Awareness: Enhanced Training for Home Health Aides* curriculum. This curriculum was created by the National Council on Aging and the Paraprofessional Healthcare Institute [17] for individuals with wide-range learning abilities and backgrounds, including nonhigh school graduates and those with limited English-language skills.

Participants in the AMOB/VLL classes were expected to attend eight 2-hour sessions led by AMOB/VLL master trainers/coaches (class sizes ranged from 3 to 18 participants with an average of 10 participants). The AMOB/VLL classes encouraged participants to (1) see falls as something that can be controlled and addressed; (2) set activity goals that gradually increase physical activity; (3) identify and eliminate falls hazards in their homes; and (4) regularly engage in exercises that increase strength and balance. The AMOB/VLL curriculum includes a participant notebook (including class content and homework), videos, various group exercises, demonstrations, and a diploma issued at the time of program completion.

The first 126 individuals who were eligible, interested, and provided informed consent were enrolled in the AMOB/VLL program. Inclusion criteria included being (1) 60 or greater years old; (2) a member of the ARC senior center and enrolled with the COASTS transportation service; (3) cognitively competent; (4) ambulatory (independently or walker/canes); (5) no contraindications to exercise as indicated by the physical activity readiness questionnaire (PAR-Q); and (6) Spanish- or English-speaking. Informed consent was obtained from all study participants, and data were collected at baseline, 8-week, 6-month, and 12-month time points. Full demographic characteristics and quantitative results from the pre-post comparison analysis are reported elsewhere [18].

### 2.2. Qualitative AMOB/VLL and COASTS Study Design

#### 2.2.1. Participant Sample

All eight MoFas and all 12 ARC master trainers/coaches who taught AMOB/VLL classes were invited to participate in focus groups. Six MoFas and five master trainers/coaches chose to participate. Master trainers/coaches attended their own focus group, and the MoFas were broken into two focus groups by primary language (Spanish-speaking: *n* = 4 and English-speaking: *n* = 2). Thirteen English-speaking, older adults participating in the AMOB/VLL classes were asked to participate in a focus group, with seven opting to do so, while 109 Spanish speaking AMOB/VLL older adult class members were invited, with 29 agreeing to participate [18]. The remaining Spanish-speaking participants were stratified into three education groups (<6^th^ grade, 6^th^–11^th^ grade, and 12^th^ grade and higher). Participants were contacted by phone by the Program Coordinator (PC). Eight participants attended the <6^th^ grade focus group, ten attended the 6^th^–11^th^ grade focus group, and eleven attended the 12^th^ grade focus group. A total of seven focus groups were conducted. Informed consent was obtained, and the study was monitored and approved by the (institution name removed for blinded review) Institutional Review Board.

Older adult focus group participants were primarily female (77.8%) and Hispanic (83.3%) with an average age of 77 years (age range 56–97). Most (61.1%) had completed middle or high school (6–12^th^ grade), 22.2% had completed primary school (0–5^th^ grade), and 16.7% had completed some college or university (>12^th^ grade). A demographic comparison of the focus group subsample is presented in Table 1.

MoFa focus group participants were primarily male (83.3%) and 50% Hispanic, with an average age of 45 years (age range 24–60 years). Four (66.7%) had completed middle or high school (6–12^th^), and 2 (33.3%) had completed some college or university (>12^th^ grade). AMOB/VLL master trainers/coaches in the focus groups were 60% female and 80% Hispanic, with an average age of 42 years (age range 28–52). One master trainer/coach (20%) reported having completed middle or high school, and the remaining four (80%) reported having completed at least some college or university (>12^th^ grade).

#### 2.2.2. Data Collection

Focus group data collection began three months after the initiation of the AMOB/VLL classes in March. MoFas participated in June, master trainers/coaches in October, and older adult program participants in December. All focus groups were held at the ARC senior center, and participants were provided transportation, if necessary. No financial incentive was provided for MoFas and master trainers/coaches. Older adult focus group participants were given $25 for participating. Prior to each focus group, the PC gave a summary of the informed consent process and asked participants to sign the informed consent document. Focus groups were led by the PC. A bilingual note-taker provided backup documentation to supplement the accuracy of audio recordings. Focus group sessions utilized a semistructured moderator's guide designed to elicit participants' thoughts on these central research questions: (1) Did participation in the course lead to positive changes in the lives of the older adult participants? (2) To what degree did the AMOB/VLL curriculum and training reflect participants' cultures, belief systems, and lifestyles? (3) What barriers and facilitators did master trainers/coaches, MoFas, and program participants encounter in relation to program implementation and the adoption of recommended approaches and exercises? All discussions were transcribed by the bilingual PC.

#### 2.2.3. Data Analysis

Focus group transcripts were uploaded into QSR International's NVivo (10.0) qualitative data analysis software, which was used to facilitate the data analysis process [19]. A thematic analysis was chosen as the analytical framework for focus group data, as it permits researchers to identify, analyze, and summarize patterns in qualitative data [20]. In the initial phase of analysis, three a priori categories were developed to capture segments of transcripts that were considered most important for addressing the primary evaluation questions. These categories included (1) positive behavior changes and outcomes; (2) cultural sensitivity and lifestyle fit; and (3) curriculum format and literacy. A fourth category, curriculum topic suggestions, was added following the initial review of transcripts, as this emerged as a prominent focus of participant discussions. For all of these categories, definitions were created to provide structure and to guide the selection and classification of codes or text segments.

Following an initial read-through of the focus group transcripts, the PC coded segments of the transcripts that related to each of the identified categories. In this process, the PC highlighted and tagged a segment of the transcript, which was then stored in the category bin(s) that most accurately captured the main idea of the transcript segment. Some transcript segments were included in several categories, as they were relevant to several of the identified categories.

Following initial sorting, data were further organized into subcategories to identify emerging themes from within larger categories. Summaries were then created for each of the categories and subcategories. Variations in dominant themes among the separate focus groups were then considered using the “coding stripes” function in NVivo. This highlighting tool coordinates the segments of transcripts that have been assigned to various categories, providing opportunities for quick, visual identification of primary themes across segments and participant groups.

## 3. Results

Program participants had overwhelmingly positive things to say about all topics, with some suggestions for curriculum improvement. Master trainers/coaches and MoFas were more mixed in their evaluation of the program's success and did not discuss every major topic.  Table 2 provides a summary from each of the focus groups (program participants, MoFas, and master trainers/coaches) for each of the four major discussion categories.  Table 3 presents specific comments for each major discussion category and provides the reader with textual examples from transcripts.

### 3.1. Behavior Change and Positive Outcomes

#### 3.1.1. Program Participants

All behavioral changes discussed by program participants were positive, and they reported being pleased with the emotional and physical benefits that resulted from participation. Benefits included a perceived reduction in falls, increased physical activity, increased confidence in their ability to take care of themselves and to be physically active, and an increased awareness of and mitigation of hazards in their environment. Participants also mentioned being happy that someone cared enough to develop and provide a program that would improve the quality of their lives, and they commented frequently on their interest in continuing the classes at their senior center. These themes were consistent across the focus groups, regardless of spoken language or education level.

#### 3.1.2. Mobility Facilitators (MoFas)

MoFas also had positive perspectives about behavior changes and program outcomes, and their comments echoed many of those made by the participants. They felt that the participants enjoyed the classes and said they believed participants would actually have preferred to have classes every day and over a longer period of time, perhaps for an entire year. MoFas also felt that the classes increased the participants' mobility and energy levels. They strongly emphasized the overall benefit of having the participants engaged in a productive and socially fulfilling activity. These themes were prominent in both the English and Spanish MoFa focus groups.

#### 3.1.3. Master Trainers/Coaches

There were few comments raised regarding changes in behavior or health outcomes in this focus group. Discussion in this group was primarily focused on curriculum content and formatting.

### 3.2. Cultural Sensitivity and Lifestyle Fit

#### 3.2.1. Program Participants

Regardless of the language or educational level, the participants agreed that the curriculum materials were culturally representative. Comments indicated that even if the individuals in the videos or handouts did not look exactly like them or have the same accent, the participants still felt connected to and represented by the individuals featured in the materials. Regarding lifestyle, various personal comments were made regarding how well the curriculum suggestions matched the participant's own abilities, with a few exceptions that included a dislike for the writing exercises or the difficulty of some of the exercises. However, in general, the comments regarding lifestyle fit were positive.

#### 3.2.2. MoFas

MoFas reported appreciating the curriculum focus on treating program participants like family and respecting them, which they felt fit well with their own personal experiences and cultural values. They further reported that the fall prevention awareness training for MoFas was beneficial for encouraging them not to rush the participants when they were getting on or off a mode of transportation. The MoFas also expressed concern about the timing of classes. The MoFas reported that participants were often worried about missing their home health aids if they went to the senior center, suggesting that creating a schedule for the participants would help them plan accordingly.

#### 3.2.3. Master Trainers/Coaches

Master trainers/coaches had the primary responsibility for interpreting the curriculum content for program participants. Master trainer/coach discussions in this category were generally critical of the degree to which materials were culturally representative, especially for Hispanic or Spanish-speaking participants. Master trainers/coaches pointed to language differences between Spanish-speaking individuals from varied locations, such as the Dominican Republic and Puerto Rico, which created communication issues. They also wanted to see more cultural diversity in the AMOB/VLL videos and felt discussions and suggestions in the materials were not easily translated to the urban setting or to the socioeconomic realities of the participants at their center living with economic disparities. One master trainer/coach also specifically raised concerns regarding a lack of direction in the curriculum for what to do when domestic abuse issues are discussed in the classroom, pointing out that it is not always accurate to assume that falls will be accidental and unexpected.

### 3.3. Curriculum Format and Literacy

#### 3.3.1. Program Participants

Regarding reading comprehension, program participants voiced a preference for larger text. In the Spanish-speaking advanced education (higher than 12^th^ grade) focus group, the participants indicated the language should be simplified and the curriculum should be adapted for individuals with different literacy levels. However, this sentiment was not echoed in any of the other program participant focus groups. The English-speaking group and the Spanish-speaking group with lower education stated that the book was appropriate for their reading and language abilities and the resources were helpful. Preferred formats for receiving information were videos, color illustrations/pictures, and class demonstrations. Participants generally preferred being active to talking about the concepts, yet they appreciated having written materials so they could review exercises and concepts. External support and approval also facilitated interest in the class, with several participants mentioning the diploma that they received and proudly sharing it with their social workers or physicians.

#### 3.3.2. MoFas

Although the MoFa discussions did not focus on curriculum formatting or literacy issues, Spanish-speaking MoFas voiced interest in having their own training curriculum translated into Spanish, so they could refresh their knowledge of the topics discussed during training.

#### 3.3.3. Master Trainers/Coaches

The master trainers'/coaches' responses in this category were critical of the curriculum and materials. They indicated that the visual aids were helpful and enabled the participants to have more productive conversations about potential hazards. However, they also stated that the individuals and scenarios presented were not always applicable to the participants' own environments, making it necessary for the master trainers/coaches to help the participants translate basic concepts to their own lives. They also felt the wording of some questions was tricky, that materials were above the reading level of some participants, and that certain Spanish translations were awkward. Differences arose between English- and Spanish-speakers regarding comprehension of concepts, with English speakers wanting explicit details on the steps necessary to reach their goals and Spanish speakers being focused on the vision of their goal rather than the details. Further, they reported homework was rarely completed, making classroom discussions difficult, with participants being reticent to participate in class if they had not completed the homework. The bulky participant binders were also identified as a mobility hazard, and the layout was considered to be awkward, requiring participants to flip back and forth. Finally, master trainers mentioned that the participants often had difficulty obtaining the signed PAR-Q (Physical Activity Readiness Questionnaire) required to attend, creating barriers to participation. One trainer stated that it could take half of a day to go to the doctor and wait in line for a signed form.

### 3.4. Curriculum Topic Suggestions

#### 3.4.1. Program Participants

Participants were largely happy with the program content. However, several participants said they would have enjoyed a more decided emphasis on balance training and techniques. They also mentioned that adding information on health problems and medications and their related impacts on balance would be helpful.

#### 3.4.2. MoFas

MoFas were satisfied with most of the topics covered in their fall prevention facilitator training, which provided numerous suggestions for getting older adults safely to and from their classes. However, they did admit that some recommendations were difficult to put into practice. For instance, they were taught to buckle the participants in on the bus and to hold their hands to help them onboard, but many participants refused their seatbelts and did not want to be touched. One topic they wanted to have added to the MoFa curriculum was a more detailed discussion of how to move participants in wheelchairs in and out of buildings, particularly in apartments that do not have ramps.

#### 3.4.3. Master Trainers/Coaches

Master trainers/coaches had numerous suggestions for curriculum content, including more physical activity and program content specifically discussing balance rather than stretching, since that is what the participants were expecting and hoping to learn. Another primary concern was the issue of fidelity, which prevented the master trainers/coaches from discussing anything not entirely related to the curriculum. They disliked the feeling of dismissing participant concerns or not exploring issues that could be relevant, particularly regarding comorbidities or medications related to balance.

## 4. Discussion

The results from our qualitative evaluation of the AMOB/VLL program combined with an ongoing COASTS MoFa program indicate participant experiences were largely positive. Benefits were also witnessed and reported by MoFas who, due to their role in participant transport, were able to witness firsthand improvements in mobility and physical activity. Although somewhat anecdotal, these outcomes are similar to those that have been previously identified with the AMOB program [12–15]. An interesting contradiction that emerged from the analysis was the difference in positive participant responses, as compared to the critical comments made by master trainers/coaches. Similar differences between participant and master trainer/coach evaluations were identified in a Dutch evaluation of a home-based AMOB program and implementation program [21]. Specifically, the master trainers/coaches in our AMOB/VLL program stated that the “one-size fits all” approach ignores the scenarios that older adults in urban minority, economically challenged neighborhoods are likely to experience. It is possible that master trainers were able to adjust their teaching strategies to provide more culturally relevant examples and advice, thereby addressing their own concerns about the curriculum themselves and providing a more positive experience for class participants.

There were several feasible adaptations suggested by focus group participants for both the AMOB/VLL and COASTS with MoFas programs. To summarize, recommendations for the AMOB/VLL program included having classes more frequently and for a longer period of time, including more cultural diversity and multiple Spanish translations for varied Spanish dialects, presenting information on falls related to domestic violence, discussing the relationship between balance and certain health problems and medications, printing materials in larger text, emphasizing balance training and techniques, and reconsidering barriers to participation (homework completion and a PAR-Q form signed by a physician). Suggestions directed towards the COASTS program included providing a schedule to participants of specific pickup and dropoff times to share with their home health aides, translating the MoFa training curriculum materials into Spanish for review purposes and providing MoFas with a longer training with a more detailed discussion of how to assist participants in wheelchairs. These adaptions and falls prevention programs will be extremely important for underserved, minority older adults who are likely to have less access to homecare and medical care resources.

### 4.1. Limitations

In this qualitative study, our focus was on gaining an in-depth, nuanced perspective of study subject experiences. This provides the opportunity to gather data that more accurately represent subject experiences. However, it typically also generates an extensive amount of subject data, meaning that sample sizes are typically small and can raise concerns over the generalizability of results. As indicated in the Methods section, our focus group participants were largely Hispanic (83.3%) and female (77.8%), with most (83.3%) of the participants completing a high school degree or less. Given these characteristics, it is questionable whether our conclusions could be translated to the larger population of aging adults residing in the US. However, as indicated in Table 1, focus group participant demographics were very similar to the larger group of AMOB/VLL participants and as such, we can assume the responses generated in the focus groups were generally representative of participants. As such, this study provides helpful guidance on how to more effectively introduce the AMOB/VLL curriculum and the COASTS mobility service in a population of urban residents. We are hopeful that these results can be further verified with additional qualitative and quantitative studies.

## 5. Conclusion

Our evaluation of an AMOB/VLL program utilized by older adults receiving the COASTS door-through-door mobility service for transportation provides a number of useful considerations for adapting future AMOB/VLL programs to fit the needs of older adults living in lower socioeconomic, urban minority environments. Potential curriculum updates that have been suggested for this population include focusing more on balance and fall mitigation strategies, including information on the impact of medications and comorbidities on balance, and adding the topic of domestic abuse. COASTS MoFas suggested coordinating transportation times with home health aides and wanted additional training and recommendations for assisting participants in wheelchairs.

Additionally, due to their role in transporting participants, MoFas could provide an excellent opportunity for conducting observation assessments of participant changes in behavior and mobility. Master trainers/coaches also appear to play a crucial role in adapting curriculum content to the literacy level and environmental realities of participants. In light of this and the many suggestions made in focus groups, we recommend including these individuals in the AMOB/VLL curriculum adjustment process. In conclusion, the AMOB/VLL curriculum provided in combination with a preexisting transportation service (COASTS) was received positively by program participants living in a low socioeconomic, urban minority community. However, additional improvements can yet be made to further encourage active participation in this evidence-based program.

## Figures and Tables

**Table 1:** Demographics comparison of qualitative subsample of older adult AMOB/VLL participants to full quantitative sample.

Characteristic	Qualitative subsample (*n* = 36)	Full quantitative sample (*n* = 126)
Mean age in years (SD)	77 (10)	74 (9)^a^
Female gender, %	77.8	78.4^b^
Education		
** **Primary school (0–5 years), %	22.2	27.6^c^
** **Middle or high school (6–12 years), %	61.1	61.8^c^
** **College/university (>12 years), %	16.7	10.6^c^
Hispanic, %	83.3	91.3

^a^ *n* = 122; ^b^*n* = 125; ^c^*n* = 123.

**Table 2:** Primary discussion points for each discussion category by participant groups.

	AMOB/VLL role	Behavior change and positive outcomes	Cultural sensitivity and lifestyle fit	Curriculum format and literacy	Curriculum topic suggestions
Program participants	Older adults participating in AMOB/VLL	**+** behavior change **+** outcomes	**+** cultural fit **+** lifestyle fit	**+** format **+** literacy	More time on balance, more active class time
Mobility facilitators	Individuals transporting participants to and from the senior center	**+** behavior change **+** outcomes	**+** cultural fit ± lifestyle fit	ND format ± literacy	Adjust advice, add info on wheelchairs
Master trainers/coaches	Individuals providing the AMOB/VLL training to participants	ND behavior change ND outcomes	**−** cultural fit **−** lifestyle fit	± format **−** literacy	More on balance, add info on medication

“**+**” indicates positive comments; “±” indicates positive and negative comments; “**−**” indicates negative comments; “ND” indicates not discussed.

**Table 3:** Notable comments made by focus group participants for each discussion category.

Discussion category	Comments by focus group participants
Behavior change and positive outcomes	*Benefits of the AMOB/VLL Course*. “I learned to walk more because I always not walking enough. Through this program I learned to walk 10 or 12 blocks every day.” (Program Participant) “This type of program keeps you moving. Some people have asked me “oh you don't have an aide?” and right way [sic] I say “I don't need an aide.” Maybe I do, but as long as I can, I will do for myself.” (Program Participant) “It [the class] always keeps them motivated with more activities. They like to be active. They always say they want to be in an activity where people feel important. If a person shows interest in a senior, that we care them…they are happier.” (MoFa) “There was this lady at the center. Her composure was like, I don't know [showed hand gesture indicating low energy]. [Moderator: “Without energy?”] Yes, without energy. She started to go to classes. [At that time] she used a cane. Afterwards, she stopped using it.” (MoFa) *Changes in Awareness of Falls Hazards*. “Through the program I also learned how to become more careful in the streets. Now I watch for cracks, I watch for the curves, how I sit down, uphill and downhill, I watch that. In the house, I took a carpet [away] in front of my sink, because I saw that was a disaster if I stumble on it and fall on my head. And then another thing, I started to do myself, I used to get up in the dark and walk around and do some things and now I turn the lights on first.” (Program Participant)
Cultural sensitivity and lifestyle fit	*Cultural Representativeness.* [Moderator asks: “In the video, you do think it was only representative of [white] Americans?” group says: “No”] “That was for everyone. Everyone that saw the videos, and saw how things are placed, that is not just for Latinos but for everyone.” (Program Participant) *Cultural Appropriateness*. “I would say, people from our country [the Dominican Republic], they like that you demonstrate love and affection. The seniors like it, well I would say all part of the world like this…I give them love, affection. Whatever senior comes to the center…or does not come to the center…That is something that I learned from my country. [Moderator: “Is that something you discussed in the training?”] Yes, because that is something that is very important…to give love to seniors.” (MoFa) “Yes, the environment is not specific or [does not] address the issue of living in a very densely inhabited, urban situation.” (Master Trainer/Coach) “For example, [one suggestion from the materials] is to make sure that there is light on when you go open the door [to the stairs]. The landlord does not fix that, there is no light there, it's dark, so you know how could they check? They don't have any control over the lighting or stairs. Some people are scared of the elevators so they take the stairs, some of the others don't live in buildings that have elevators. They cannot control the environment.” (Master Trainer/Coach) *Timing of Classes*. “[If the aide has not shown up] then they won't go to class. They say “no, because I will lose my home attendant” and that is a big excuse. They should vary the schedule. Maybe classes should start later at 10 a.m. so they have more time.” (MoFa 1) “Yes, there are many people who say “Oh, I have not eaten anything. My home attendant she is the one who prepares breakfast.” (MoFa 2) *Domestic Abuse*. “I feel that the book never took into account that people were just going to fall because of an accident [due to] something that was not expected. I don't think the study itself works with people that have been abused, who have additional psychological problems…The people [in the] original part of the study never brought [these issues] to the table because it was never part of their living experience, unlike most seniors who have huge negative experiences because of economic factors.” (Master Trainer/Coach)
Curriculum format and literacy	[Moderator: “Was there anything that affected your learning? Was there anything that was confusing or not clear?”] “Everything was clear.” (Spanish speaker participant, <6^th^ grade education) “Very clear.” (Spanish speaker participant, <6^th^ grade education) “In the class there were many times that happened [having written materials to use at home and refreshing their memories about certain exercises or concepts]. [Master Trainer/Coach A] and [Master Trainer/Coach B] they were doing the exercises and we would copy them. Also, there was a class where we were the instructors.” (Program Participant) “I thought that they were all very easy, at the time when I went home I looked at the illustrations. I Wanted to sort of review and go to illustrations and try stuff out.” (Program Participant)” “People would actually say… “Pero esa pregunta, es muy estranga, esa pregunta no la entriende nadie.” [The question I know…it is very strange, that question nobody understands it.]” (Master Trainer/Coach) “It was an interesting dynamic how much effort was put in to making a plan [for the English group] compared to the other group [Spanish] who were like “this was a waster of my time, this was too much effort.” (Master Trainer/Coach)
Curriculum topic suggestions	“I would like to see more balance in the class.” (Program Participant 1) “[I would like more information about] techniques because I have a problem with balance, just like if I start this way and all of a sudden, I have to back up, I have to always think, “Don't turn around so fast because you will lose your balance.” I would like to see more balance, how to focus more.” (Program Participant 2) “There's a lot of medical issues, things involved here and we are not prepared. There's a lot of joint…and, even, I'm prepared to do the muscular…you know, the physical, the joint, all that conversation, but about medication I don't…about mental disease, I don't. I Know very little about insulin, and sugar deficit and all that…I don't know anything. I Felt like I was not prepared to discuss that, as part of the fear of falling.” (Master Trainer/Coach 1) “So a lot of our participants wanted to know what their medicines, how those medicines were impacting them, because a lot of them felt that also some of the stuff that they were taking for medicinal purposes, were affecting their balance. Whether it's true or not true…there's no way to address that.” (Master Trainer/Coach 2)

## Data Availability

The qualitative data used to support the findings of this study are restricted by the Columbia University Institutional Review Board in order to protect research subject privacy. Data are available from Thelma Mielenz at the Mailman School of Public Health, Columbia University, 722 West 168th Street, New York, NY 10032, for researchers who meet the criteria for access to confidential data.
