# Corrigendum to “Fall Risk Reduction Program Paired with a Transportation Program in an Underserved, Urban Minority Community: A Qualitative Evaluation”

**DOI:** 10.1155/2020/4721683

**Published:** 2020-10-13

**Authors:** Thelma J. Mielenz, Laura Durbin, Fern Hertzberg, Diana Noble-Hernandez, Julie A. Sorensen

**Affiliations:** ^1^Mailman School of Public Health, Columbia University, 722 West 168th Street, New York, NY 10032, USA; ^2^ARC XVI Fort Washington Inc. (ARC), 4111 Broadway, New York, NY 10033, USA; ^3^Bassett Research Institute, One Atwell Rd., Cooperstown, NY 13326, USA

In the article titled “Fall Risk Reduction Program Paired with a Transportation Program in an Underserved, Urban Minority Community: A Qualitative Evaluation” [1], the Acknowledgements section was omitted from the article in error. The acknowledgements section should read as follows:

“This research is supported by Grant 1 R49 CE002096-01 from the National Center for Injury Prevention and Control, Centers for Disease Control and Prevention to the Center for Injury Epidemiology and Prevention at Columbia University. Its contents are solely the responsibility of the authors and do not necessarily represent the official views of the Centers for Disease Control and Prevention. We dedicate this manuscript to Diana Noble-Hernandez for her tireless efforts in supporting older adults in their communities across Upper Manhattan.”
